# Set screw fracture with cage dislocation after two-level transforaminal lumbar interbody fusion (TLIF): a case report

**DOI:** 10.1186/1752-1947-9-22

**Published:** 2015-01-21

**Authors:** Philip Johannes Felix Leute, Ahmed Hammad, Isabel Hoffmann, Sebastian Hoppe, Hans-Michael Klinger, Stefan Lakemeier

**Affiliations:** Abteilung für Orthopädie, Universitätsmedizin Göttingen, Robert-Koch-Strasse 40, 37075 Göttingen, Germany

**Keywords:** Complication, Fracture, Set screw, TLIF, Transforaminal lumbar interbody fusion

## Abstract

**Introduction:**

Transforaminal lumbar interbody fusion is a popular procedure used to achieve spondylodesis in patients with degenerative lumbar spinal diseases. We present a rare case of a patient with a set screw fracture with cage dislocation after an open transforaminal lumbar interbody fusion procedure. To the best of our knowledge, this case is the first of its kind to be reported.

**Case presentation:**

A 44-year-old Caucasian woman attended a follow-up appointment at our hospital 3 months after treatment for second-degree lumbar spondylolisthesis (L4/L5) and osteochondrosis (L5/S1) with transforaminal lumbar interbody fusion and dorsal spondylodesis. She complained of severe leg pain on the left side. Her physical examination revealed a normal neurological status, except for paresthesia of the entire left lower limb and at the ball of the left foot. Radiological imaging showed breaking of the set screws with cage dislocation. Surgical revision was then performed with exchange of the whole dorsal instrumentation and the dislocated cage. Six weeks post-operatively, the patient was seen again at our clinic without neurological complaints, except for decreased sensitivity on the dorsum of her left foot. The wound healing and radiological follow-up were uneventful.

**Conclusions:**

Hardware-related complications are rarely seen in patients with open transforaminal lumbar interbody fusion, but must be kept in mind and can potentially cause severe neurological deficits.

## Introduction

Transforaminal lumbar interbody fusion (TLIF) is a popular procedure used to achieve spondylodesis in patients with degenerative lumbar spinal diseases [1]. Neurological, wound-healing and hardware problems have been reported in these patients [2]. Nevertheless, hardware problems with implant fractures are a rare complication of the procedure [3]. We present a case of implant fracture with cage dislocation after an open TLIF procedure. To the best of our knowledge, this is a complication after open TLIF that has not been reported in the literature to date.

## Case presentation

**Figure 1:**
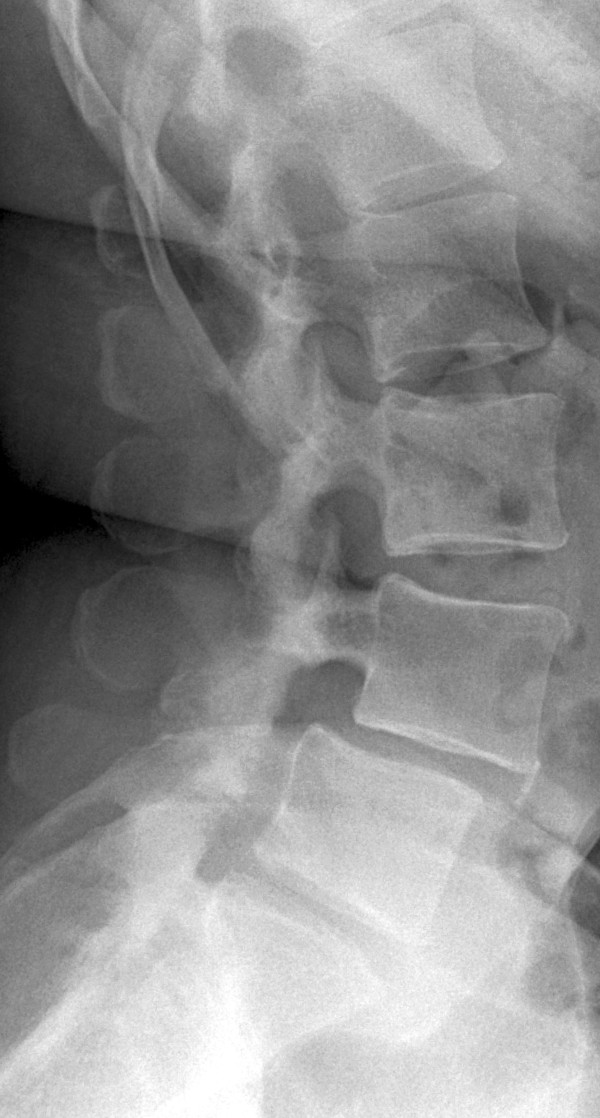
**X-ray taken before the patient’s first surgery.**

**Figure 2:**
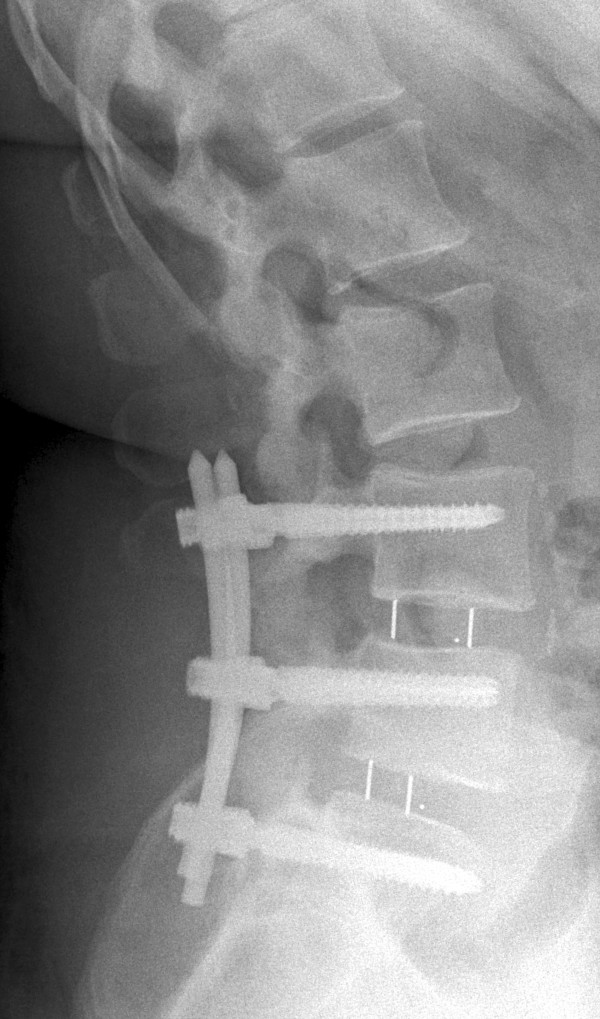
**X-ray obtained after the patient’s first surgery.**

A 44-year-old Caucasian woman came to our outpatient clinic for a 3-month follow-up appointment after undergoing an open two-level (L4-S1) TLIF procedure with dorsal spondylodesis. She had been treated for spondylolysis with second-degree lumbar spondylolisthesis (L4/L5) and for osteochondrosis (L5/S1) (Figures 1 and 2). At her follow-up visit, she complained of pain in her whole left leg.

A physical examination revealed pain upon palpation of the lumbar spine. The patient’s spinal muscles were tense. Intermittent paresthesia of her left lower limb and constant paresthesia at the ball of the left foot were present. There was no saddle block paresthesia. Her motor function was MRC (Medical Research Council) Scale 5/5 in all muscles of both legs. Her Lasègue and Bragard signs were positive on the left side. Her bilateral patellar and Achilles tendon reflexes were regular, as was her perianal sensitivity and sphincter muscle tone. No urinary or defecation problems were observed. The patient was in good general condition, but overweight (108 kg weight, 170 cm height). She had sustained no trauma. She had been compliant with the given instructions for sitting and working in a manner that is easy on the back. However, she had not lost any body weight as we had advised.

Her past medical history included arterial hypertonus, restless legs syndrome and an axial esophageal hernia. Her past surgical history consisted of a hysterectomy and tonsillectomy, 6 and 31 years ago, respectively. Upon admission to the hospital, she was taking several painkillers, blood pressure medication, a dopamine agonist and a proton pump inhibitor.

**Figure 3:**
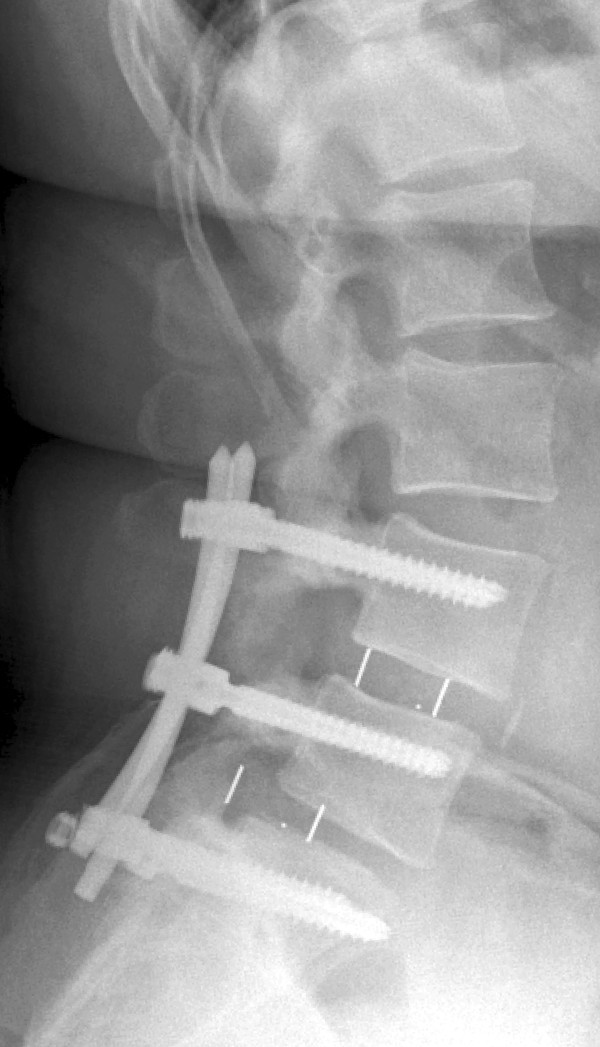
**X-ray taken at first follow-up examination.**

**Figure 4:**
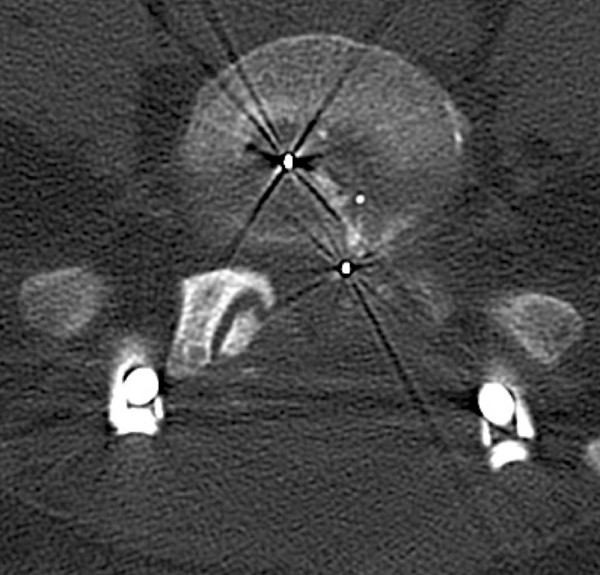
**Computed tomographic scan taken at first follow-up examination.**

Her social history revealed that she had consumed nicotine since she was 14 years old (46.5 pack-years). Her family history and review of her organ systems were non-contributory, except for the above-mentioned diseases.Radiography and computed tomography were performed (Figures 3 and 4). A diagnosis of implant failure with a combination of cage dislocation left dorsally and breaking of the set screws on both sides with dislocation of the rods was made. All polyaxial screws were intact and in place, except for the S1 screws, which had become loose and dislocated dorsally on both sides.

**Figure 5:**
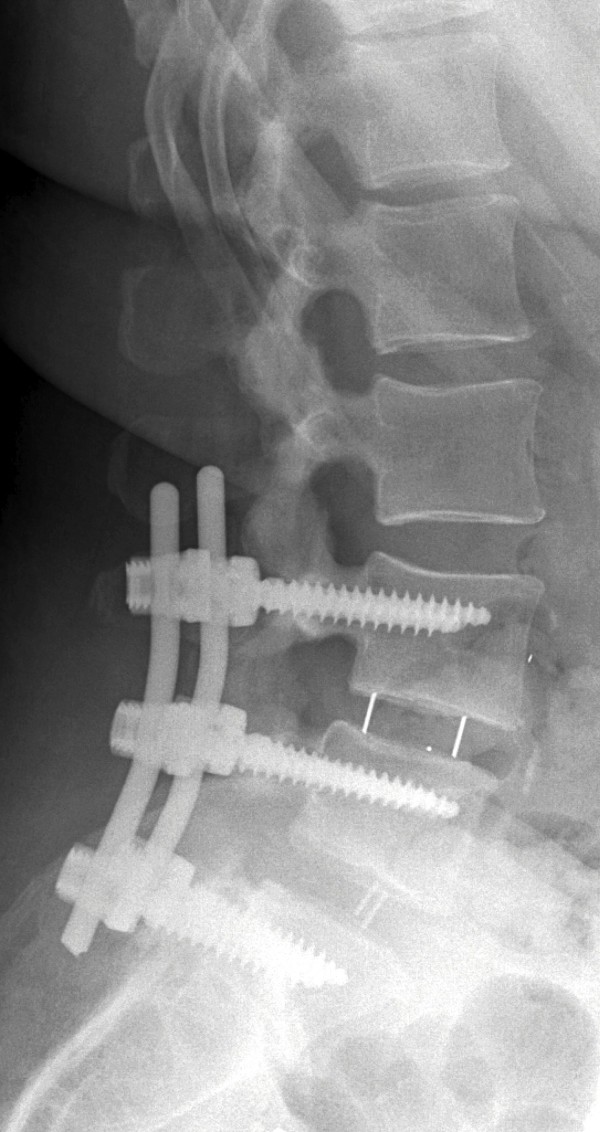
**X-ray taken after the patient’s second surgery.**

**Figure 6:**
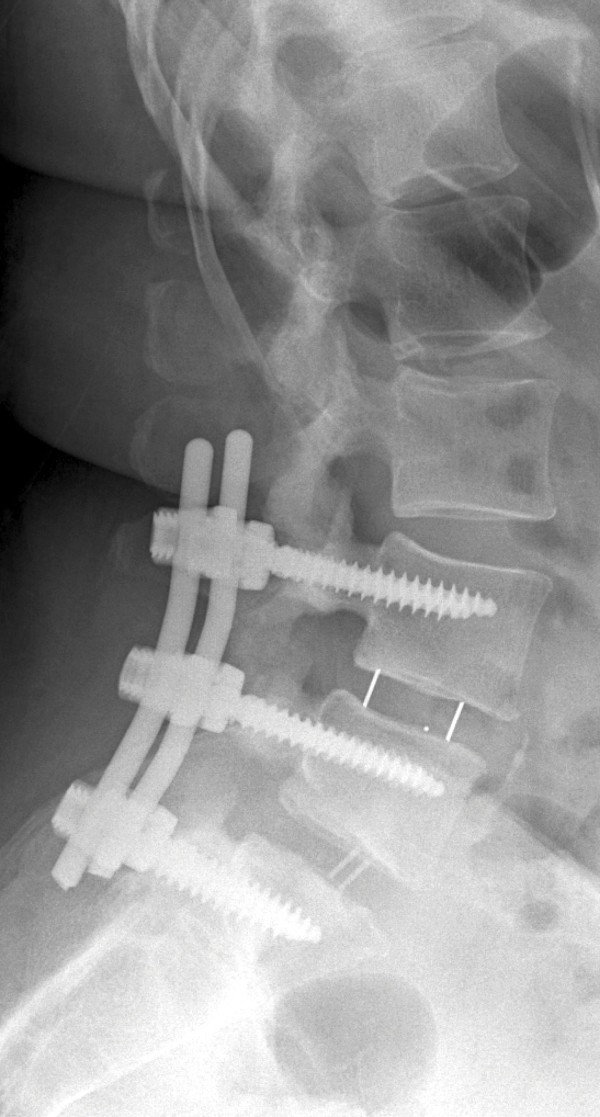
**X-ray taken at the patient’s second follow-up examination.**

A revision surgery was performed by the same surgical team that had done the primary operation. The patient was placed in prone position. A midline incision was made through the existing scar. Removal of cicatricial tissue was performed until all of the screws could be exposed. The S1 polyaxial screws were loose and dislocated on both sides. Metallosis was observed in the surrounding tissue. The S1 set screws were then inspected and removed. They showed a breaking of the worm. The other polyaxial screws were in place and intact. The L5 nerve root was prepared, followed by exposure of the dura. From there, preparation toward the cage in the L5/S1 intervertebral disc space was done. Palpation showed that it had become completely loose. The cage was removed. Next, expansion of the intervertebral disc space, rasping of the edges and insertion of a new cage was done. After that, the whole dorsal instrumentation system was exchanged. Pedicle screws with a 0.5-mm-larger outer diameter than those previously used were placed. Finally, the wound was closed layerwise, and two drains were placed. No intra-operative complications occurred.A post-operative X-ray showed a regular position of the implants (Figure 5). The drains were removed on the second post-operative day. The wound healing was primary and without signs of infection. The patient’s neurological complaints (pain and paresthesia) disappeared completely. Again, physiotherapists instructed the patient to sit and work in a manner that is easy on the back, and she was advised to lose weight and quit smoking. Twelve days after the operation, the patient was discharged to home. From there, she went into a rehabilitation clinic. No peri-operative complications occurred.Six weeks post-operatively, the patient was seen at our outpatient clinic for a follow-up examination (Figure 6). She complained of back pain without radiation to the legs, which was controllable with pain medication. In addition to that, she complained that the dorsum of her left foot had decreased sensitivity. A physical examination revealed a regular scarring of the wound. Paresthesia was present on the dorsum of her left foot. Apart from that, sensitivity was the same bilaterally. Her motor function was MRC (Medical Research Council) Scale 5/5 in all muscles of the legs bilaterally, and she had regular reflexes. Her Lasègue and Bragard signs were negative bilaterally. No post-operative complications were observed.

## Discussion

To identify similar cases, we reviewed the online databases PubMed, MEDLINE, CareLit and MEDPILOT for the keywords "TLIF," "transforaminal lumbar interbody fusion" and "complication." The search revealed 244 publications. We excluded 204 studies that covered inappropriate topics. This left us with 40 studies with comparable patients and treatments. Implant-related complications were found in 22 of these studies and were present in 76 cases (Table 1) [1–22].

**Table 1:** **Review of the literature**

Year	Author	Hardware failure not specified	Cage migration	Screw loosening or backing out	Screw or cage malposition	Cage subsidence	Rod fracture
2004	Castro *et al*. [4]	0	0	0	6	0	0
2005	Hackenberg *et al*. [5]	0	0	1	0	0	0
2006	Lauber *et al*. [6]	0	0	1	0	0	0
2006	Weiner *et al*. [7]	1	0	0	0	0	0
2006	Taneichi *et al*. [8]	0	1	1	0	0	0
2008	Yan *et al*. [9]	0	0	1	0	0	0
2009	Xu *et al*. [10]	0	2	1	0	0	0
2009	Faundez *et al*. [11]	0	0	0	2	0	0
2010	Gong *et al*. [12]	0	0	1	0	0	0
2010	Fujibayashi *et al*. [13]	0	0	0	1	0	0
2010	Wang *et al*. [14]	0	0	0	1	0	0
2011	Mura *et al*. [15]	0	3	0	5	3	0
2011	Takahashi *et al*. [2]	0	4	0	0	0	0
2012	Burneikiene *et al*. [3]	0	0	3	0	0	2
2012	Aoki *et al*. [16]	0	1	0	0	0	0
2012	Kim *et al*. [17]	0	1	0	0	0	0
2012	Lee *et al*. [18]	0	6	0	0	0	0
2012	Tormenti *et al*. [1]	0	10	0	11	0	0
2013	Høy *et al*. [19]	0	0	0	2	0	0
2013	Lau *et al*. [20]	0	0	0	2	0	0
2013	Seng *et al*. [21]	0	2	0	0	0	0
2014	Zhang *et al*. [22]	0	0	0	1	0	0

Of these hardware-related complications, 75 were further specified and 1 was unspecified. There were 31 cases with cage or pedicle screw malpositioning, 30 with cage migration, 3 with cage subsidence, 9 with pedicle screw loosening or backing out and 2 with a rod fracture. However, none of these cases were comparable to the one described in the present report, which uniquely consisted of loosening of the polyaxial sacral screws, fracturing of the set screws and cage dislocation.

Although our report provides no causal explanation for this kind of implant failure, we assume that the size and placement of the implant did matter in our patient. Furthermore, we believe that the dorsal fixation device may have been too small for our patient’s biomechanical needs. Cho and coworkers reported that pull-out strength of the pedicle screws increased with the outer diameter of the screws [23]. Therefore, we assume that our primary choice to use pedicle screws with a smaller outer diameter led to early loosening of the sacral screws in our patient. Only after exchanging the primary system for a larger one did we achieve good biomechanical stability.

The broken set screws make the present case unique. We could not identify any matching cases that have been reported to date. However, in a similar report, Agrawal described a case of nut loosening, and thus a loosened set screw after dorsal instrumentation, but without breakage of the worm [24]. Moreover, nut loosening was reported by Davne and Myers for dorsal instrumentation and posterior lumbar interbody fusion with a variable screw placement system, which uses a plate over which screws are placed into the bone [25]. In their large series of 486 patients, they attributed a certain amount of surgical inexperience to most cases of nut loosening. In our case, we assume that it was not inexperience of the operating surgeons that led to breakage of these screws. However, we acknowledge that, in general, malpositioning of the set screws or flaws in their production can lead to a certain predisposition for the worm to break.

With regard to fixation of the pedicle screws, Ha and coworkers showed that side fixation of the pedicle screws led to greater stability than head fixation with a set screw [26]. We were unable to identify any literature addressing whether an increase of the contact face of the set screws also leads to greater stability. Nevertheless, in our patient, the exchange of the internal fixation system led to the implantation of a set screw with a larger contact face. Whether an increase of the contact face of the set screws leads to greater stability remains unclear.

Cage migration typically occurs dorsally, as it did in our patient [1]. It is also associated with cage placement in a central position as compared to cages placed in anterior positions [27]. In our patient, the migrated cage between L5 and S1 lay rather dorsally. This may have also predisposed the cage to migrate in this direction.

With regard to our patient’s obesity, the available literature shows no difference in operative time, length of hospitalization or complication rates in general between obese and non-obese patients [28]. Nevertheless, this remains a controversial topic, and therefore obesity must be kept in mind as a possible negative factor.

The fact that our patient has many pack-years of smoking is unlikely to have contributed to her outcome. Our review of the literature showed that negative effects of smoking in TLIF have not been observed and can probably be excluded as a factor related to poor outcome [1, 2, 20].

## Conclusions

The complications we describe in this report are particularly of interest for spinal surgeons. To the best of our knowledge, this report is currently the only available one of its kind in the literature.

The following take-home messages must be taken into account. (1) Hardware-related problems are a rare complication in open TLIF, but must be kept in mind. (2) Hardware-related problems must be mentioned when obtaining informed consent from a patient prior to performing open TLIF. (3) Hardware-related complications can potentially cause severe neurological deficits. (4) Lifestyle modifications should be advised for all patients, if appropriate.

## Consent

Written informed consent was obtained from the patient for publication of this case report and any accompanying images. A copy of the written consent is available for review by the Editor-in-Chief of this journal.

## Abbreviations

TLIF: Transforaminal lumbar interbody fusion.
